# Proactive Parent Engagement in Public Schools: Using a Brief Strengths and Needs Assessment in a Multiple-Gating Risk Management Strategy

**DOI:** 10.1177/1098300716632590

**Published:** 2016-03-04

**Authors:** Kevin J. Moore, S. Andrew Garbacz, Jeff M. Gau, Thomas J. Dishion, Kimbree L. Brown, Elizabeth A. Stormshak, John R. Seeley

**Affiliations:** 1 University of Oregon, Eugene, USA; 2 Oregon Research Institute, Eugene, USA; 3 Arizona State University, Tempe, USA

**Keywords:** family engagement, multiple gating, positive behavior support

## Abstract

This study examined the viability of a brief, parent-reported strengths and needs assessment as the first step in a multiple-gating approach to proactive positive behavior support for families. The *Positive Family Support–Strengths and Needs Assessment* (PFS-SaNA) was designed to collaboratively engage parents early in the school year in a home–school coordinated Positive Family Support (PFS) system. In this study, we evaluated the reliability and validity of the PFS-SaNA in the context of public middle schools. Findings suggest that the 14-item, unidimensional PFS-SaNA shows convergent validity with teacher ratings of risk. It can be easily and cost-effectively used by school personnel when parents register their children for school at the beginning of each school year.

Reviews of the literature suggest “consistent, positive, and convincing” evidence for the positive effects of parent engagement in children's education (Henderson & Mapp, 2002, p. 7). *Parent engagement in education* refers to behaviors that are “interactive, purposeful, and directed” (Sheridan, Knoche, Kupzyk, Pope Edwards, & Marvin, 2011, p. 362) and that support youth by integrating parenting support within the school context to improve student academic outcomes and reduce problem behavior (Dishion & Kavanagh, 2003). Parent engagement interventions in middle school have been shown in randomized, controlled trials to reduce negative outcomes, such as early adolescence drug use (Dishion, Kavanagh, Schneiger, Nelson, & Kaufman, 2002; Stormshak et al., 2011), depression (Connell & Dishion, 2008), antisocial behavior (Van Ryzin & Dishion, 2012), and negative peer affiliation (Van Ryzin, Stormshak, & Dishion, 2012), while positively influencing student self-regulation, grade point average, attendance, and family dynamics (Fosco, Frank, Stormshak, & Dishion, 2013; Stormshak, Connell, & Dishion, 2009).

Despite empirical evidence linking parent engagement with improvements in student adjustment, many tiered systems of student support in schools, such as Response to Intervention (RtI) and School-Wide Positive Behavioral Interventions and Supports (SWPBIS), have only minimally addressed the shared responsibility educators and families have to educate children. Specifically, families' participation in their children's education most frequently involves selected or indicated interventions after significant concerns have become apparent in the school setting (Fox, Dunlap, & Cushing, 2002). There are two limitations to this practice. First, missed opportunities to proactively manage and address students' unique needs may lead to the development of more serious problems. Furthermore, parents may experience stigmatization when their youth is defined as “at risk” in the absence of a specific incident (see the “Multiple Gating” section).

The second limitation is that assessments of a student's behavior only at school may be less valid than those that capture variation in behavior at home and at school. For example, youth who have challenges at home and at school show much deeper adjustment problems and higher rates of future difficulties than those who have concerns at either home or at school (Dishion, 2000). Unfortunately, clear procedural recommendations for engaging families in universal interventions for addressing social behavior often do not exist even though several parent-report screening forms are available (e.g., Kamphaus & Reynolds, 2007).

## Multitiered Positive Family Support (PFS)

A public health perspective is advisable when designing and implementing school-based behavioral health interventions (Dowdy, Ritchey, & Kamphaus, 2010; Shaw, 1986). Schools are the largest provider of child behavioral health services and for many children, the only community environment where they will receive behavioral health intervention (Hoagwood, Burns, Kiser, Ringeisen, & Schoenwald, 2001). Evidence suggests that the rates of childhood emotional and behavioral problems can be efficiently reduced if families and children are engaged at the tier of support appropriate for their needs (Dishion & Kavanagh, 2003; Dishion & Stormshak, 2007; Horner et al., 2009). Thus, behavioral health services that are school based and family focused have the potential to engage large numbers of individuals and significantly reduce the prevalence of behavioral health and learning needs (e.g., Miller, Brody, Yu, & Chen, 2014).

The PFS approach (Dishion, 2011; Fosco et al., 2014) is based on a 25-year program of research on the integration of evidence-based family interventions into the school context to improve student academic engagement and to reduce problem behavior and drug use. Three principles were derived from the series of studies, as were developments in educational interventions. First, family intervention must be integrated into a broad, school-based system of behavioral support (Dishion, 2011). Second, family interventions can be brief, motivational, and adapted to the specific needs of children and families (Dishion & Stormshak, 2007). Third, family interventions will be most effective when deployed proactively, as opposed to reactively, in the school context (Christenson, 2004). It is thought that applying these three principles into strategies for parent engagement will lead to the translation of evidence-based parent support into the daily operations of a school system.

Thus, PFS was specifically designed to be coordinated within tiered, school-wide behavior support programs, such as SWPBIS, by linking comprehensive family connections at every tier. For example, the universal level comprises the *Positive Family Support–Strengths and Needs Assessment* (PFS-SaNA) and a staffed family resource center that offers informational brochures, flyers, and video examples of proactive parenting skills, such as positive behavior support at home, limit setting, monitoring of peers and schoolwork, getting to bed on time, and home–school connections. Staff development strategies aim at increasing family outreach, increasing positive parent contact and communication before behavior problems take root, and targeting positive communication strategies about attendance, schoolwork, and behavior. At the second tier, home-based incentives and parent–child interaction strategies are developed that endorse school-based interventions and strategies, such as Check-In/CheckOut (Todd, Campbell, Meyer, & Horner, 2008), behavior change plans, and support for homework completion and passing specific subject proficiencies. The Family Check-Up (Dishion & Stormshak, 2007), at the high second and third tiers, includes tailored follow-up parenting support that is assessment driven and menu based and that dovetails with a school-based functional behavioral assessment and intervention plan. PFS was designed to cohere with Positive Behavioral Interventions and Supports (PBIS) because PBIS is considered the most scaled-up, evidence-based practice in the human services field (Blasé et al., 2008).

## Screening Within a Multitiered Model

### Multiple Gating

Multistage assessments have been widely used and found to be economically feasible and valid for making decisions about hiring personnel (Cronbach & Gleser, 1965). The multistage principle is to use the least expensive assessments at Stage 1 to refine the pool of potential applicants and to save the more expensive assessments for the final selection process with a smaller group of applicants. This basic idea has been applied when identifying high-risk youth in public schools (Dishion & Patterson, 1993). It was originally proposed that *Teacher Risk Assessments* (TRISKs) be used first, parents assessments second, and then more intensive family management assessments at the third gate. This strategy was used in the original prevention trial of the Family Check-Up (Dishion & Kavanagh, 2003). A similar strategy was developed that involved only school-level data, with teacher nominations followed by direct observation of the student in the school setting (Walker & Severson, 1992). This school-based, multiple-gating strategy was successfully used and widely disseminated; however, the strategy did not include parent screening assessments.

**Figure 1:**
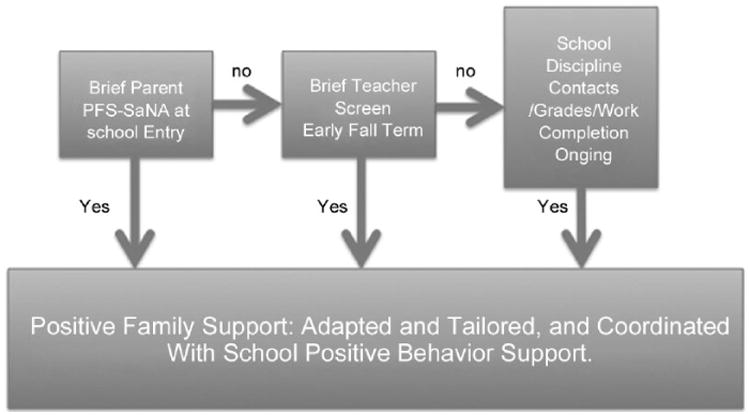
Proactive parent engagement multiple-gating approach to risk management.

For researchers interested in developing empirically validated family supports, it quickly became apparent that the sequencing of teachers as the first gate was psychometrically efficient but unfortunately became a barrier to subsequent engagement of parents in the risk assessment and intervention process (see Dishion & Kavanagh, 2003). About 50% of the identified parents declined to participate. Informal perusal of the parents' refusal rationale revealed that stigmatization was high on the list of reasons, especially for families of color. In addition, previous studies had revealed that the overall predictive efficiency was the same regardless if one began with parents or teachers in the multiple-gating assessment process (cf. Dishion & Patterson, 1993). Therefore, while designing the PFS proactive assessment and engagement process, we hypothesized that barring logistical challenges, all parents could complete a readiness screen at school entry if the wording of the items were respectful and clearly linked to concerns parents might have about their youth during transition to public middle school. We therefore revised the approach, as shown in Figure 1.

The many limitations of existing population-based behavioral assessment in schools have prevented broad implementation (Dowdy et al., 2010; Lane, Menzies, Oakes, & Kalberg, 2012), and these limitations increase for middle and high school contexts. One important limitation is that implementation guidelines lack recommendations to increase family engagement. Another primary limitation is that family management (i.e., positive support, limit setting, monitoring, problem solving) and peer involvement (i.e., positive or antisocial peer groups; Dishion & Stormshak, 2007), two of the most important contextual factors associated with behavioral problems, often are not assessed. That is, current assessment/screening processes are decontextualized from the proximal variables (i.e., parenting practices and peer associations) most associated with positive or negative life course outcomes in this age group, without any pragmatic method to recontextualize them for intervention purposes.

More than a half century of evidence suggests that the middle school years and early adolescence are a critical developmental transition that if successfully navigated by families and students, augurs well for youths' positive life course developmental outcomes (Eccles & Harold, 1996; Lane, Oakes, Carter, & Messenger, 2015). Thus, middle school is not only a key developmental period but also a key ecological setting in which to develop and execute a systematic public health model of behavioral health service delivery that proactively engages parents. To date, there are no publications about brief and positively worded screening measures for parents who are specifically linked with school-wide initiatives (e.g., SWPBIS) in middle schools.

### PFS-SaNA

The PFS-SaNA was developed in response to the need for an assessment that can increase family engagement in middle school and assess contextual factors associated with proximal variables and linked with behavior concerns. The historical sequencing of the multiple-gating strategy (i.e., teachers in the first gate, parents in the second) was reordered to promote parent engagement as a potential alternative to current universal approaches. Using parents as informants during initial assessments may help address the barriers and future research needs cited by others (e.g., Dowdy et al., 2010). The PFS-SaNA asks parents about students' strengths and needs and need for support in the assessed areas. This approach provides a systematic mechanism for proactive parent contact before significant concerns arise. Proactive parent contact has been recommended for years; however, we could find no examples in the literature of a school-based model that advocates for, or uses, parents as a primary source of systematic assessment information in middle schools, that specifically focuses on the universal tier, and that is linked to proximal attributes of student functioning. Systematic, proactive parent participation provides a critical home–school link in family-centered models of prevention (Dishion, 2011; Fosco et al., 2014). As shown in Figure 1, a suggested reordered timeline would be as follows: PFS-SaNA is collected before the school year begins (e.g., during registration). Then, during the second month and midyear of school (e.g., October, February), a brief teacher screen is administered, and ongoing data teams analyze the results along with additional school-based screening measures (e.g., attendance, discipline contacts).

## Purpose of the Study and Research Questions

The purpose of this study was to psychometrically evaluate the PFS-SaNA for use in middle schools within a public health framework. The following specific research questions were examined:

**Research Question 1:** What is the factor structure of the PFS-SaNA for a middle school sample?

We hypothesized that the PFS-SaNA would be reliable and produce a one-factor solution indicating that the screener is unidimensional, consistent with other screening systems (Kamphaus & Reynolds, 2007).

**Research Question 2:** What is the technical adequacy of the PFS-SaNA in middle schools?

We hypothesized that the PFS-SaNA would moderately to highly correlate with social and emotional behavior scales.

## Method

### Participants and Setting

Participants were parents and teachers of middle school students at eight participating middle schools (Grades 6–8) in the Northwest region of the United States. The schools had at least begun to use the PFS-SaNA during the first 2 years of a larger multiyear, multicohort implementation trial. Parents were involved in the larger study through an opt-out procedure, and all middle schools in the target region were invited to participate. Schools that agreed to participate were then randomly assigned to a wait-list control or the larger intervention.

Analyses occurred in two phases. Phase 1 included an exploratory factor analysis (EFA) of the PFS-SaNA, based on 448 students. Phase 2 included a confirmatory factor analysis (CFA) and an examination of technical adequacy, which was based on 422 students. In Phase 1, 50% of the students were female, and the average age of the group was 12.90 years (*SD* = 0.80). Of the 50% of students who reported their ethnicity, 66% were European American, 14% were American Indian/Alaskan Native, 3% were Asian, 2% were African American, 5% identified as Other, and 10% reported multiple ethnicities. As an indicator of socioeconomic status, students responded to the question, “How much money does your family have?” Forty-five percent of students reported *just enough to get by*, 38% reported that they *only have to worry about money for fun and extras*, 13% indicated *we never have to worry about money*, and 4% reported *not enough to get by*. In Phase 2, 53% of the students were female and the average age of the group was 11.8 years (*SD* = 0.6). Of the 60% of students who reported their ethnicity, 67% were European American, 10% were American Indian/Alaskan Native, 1% were Asian, 2% were African American, 8% identified as Other, and 12% reported multiple ethnicities. Forty-five percent of students reported their family had *just enough to get by*, 34% reported that they *only have to worry about money for fun and extras*, 16% indicated *we never have to worry about money*, and 4% reported *not enough to get by*.

Of the participating schools, 75% were located in rural settings and 25% were located in urban or suburban settings. The number of enrolled students per school ranged from 290 to 756 (*M* = 487). The percentage of ethnic minority students ranged from 15.2% to 54.9% (*M* = 30.3%), and the percentage of students who qualified for free or reduced-price lunch ranged from 48% to 91.8% (*M* = 63.2%). Table 1 presents the total number of PFS-SaNAs completed at each school, the proportion of eligible students at each school whose parents completed a PFS-SaNA that could be matched to participant data, and a description of the matching procedure.

### Measures

#### Parent-report measures

The PFS-SaNA was adapted from the TRISK (Soberman, 1994) and the *Secondary School Readiness Inventory* (SSRI; created by the study authors). In a methodological study, the TRISK demonstrated adequate psychometric properties (Soberman, 1994). In this sample, the adapted TRISK demonstrated good reliability (Cronbach's α = .91) and the SSRI demonstrated good reliability (Cronbach's α = .92). Adaptation and development of the PFS-SaNA were completed by a panel of experts in school-based mental health prevention and intervention, multiple gating, and screening (i.e., three research scientists with more than 60 years of combined experience developing measures and interventions in these topic areas). The PFS-SaNA is designed for completion by parents/primary caregivers of middle school students. Each parent/caregiver rates their student on 14 items about common areas in which students may need additional support (e.g., avoiding difficult or challenging tasks; being depressed, anxious, or irritable). Each item is rated on a 3-point Likert-type scale in one of three categories to reflect a parent's concern for his or her child (i.e., *doing great*, *some concern*, *serious concern*). The scale score is the summed total. In addition, parents can indicate via a checkbox whether they think their child needs support for each item. The PFS-SaNA was completed in the fall on paper-based forms.

Thirteen of the 14 items on the PFS-SaNA were included in a parent questionnaire that was completed in the summer. Six items were drawn from an adapted version of the TRISK (Soberman, 1994) and included as a parallel parent-report form. The adapted TRISK consisted of a 4-point Likert-type scale ranging from 0 (*no concern*) to 3 (*serious concern*). The remaining seven items were drawn from the SSRI. The SSRI consisted of a 10-point Likert-type scale ranging from 1 (*needs support*) to 10 (*area of strength*). Total scores were calculated separately for the adapted TRISK and SSRI. To calculate a correlation between these items and the PFS-SaNA, the two total scores were standardized to *z* scores and summed to create an overall score congruent with the total score of the PFS-SaNA. To facilitate item-level comparisons across items on the adapted TRISK and PFS-SaNA, the response options of *mild concern* and *moderate concern* were recoded to match the PFS-SaNA response, *some concern*.

To determine whether school personnel's contact with parents was related to parent report of concerns on the PFS-SaNA, the following two parent-report items were examined: How often has someone from the school contacted you about your child's negative behavior? How often has someone from the school contacted you about your child's positive behavior? Both items were rated on a 4-point Likert-type scale ranging from 0 (*never*) to 3 (*weekly or more*). These two items were completed by parents in the summer as part of a paper-based assessment. Screening procedures for the PFS-SaNA are described in the “PFS-SaNA Procedures” section.

#### Teacher-report measures

Measures completed by teachers were administered in the spring via an Internet-based software system. On the *Teacher Concern for Student School Adjustment Scale* (TCSSAS), teachers rated the extent to which they were concerned with students in six areas/items (i.e., depressed, anxious, or irritable; relations with other students; spends time with students who break school rules; follows classroom rules; stays on task; completes homework and assignments on time) on a 4-point Likert-type scale ranging from 0 (*no concern*) to 3 (*serious concern*). The TCSSAS was developed for our study and is a modified version of the TRISK (Soberman, 1994). To create the TCSSAS, the items on the TRISK were modified so all were stated positively and aligned with the other parent-completed measures. The TCSSAS was collected from teachers in the spring. The TCSSAS demonstrated good internal reliability with the study sample (Cronbach's α = .91). The TCSSAS was examined as a total concern scale score, and items on the TCSSAS that aligned with items on the PFS-SaNA were evaluated. Because the response options across the TCSSAS and PFS-SaNA differed, responses of *mild concern* and *moderate concern* on the TCSSAS were recoded to match the PFS-SaNA response, *some concern*.

#### Student-report measures

Students completed the aforementioned indicator of socioeconomic status. Students also responded to one item that asked, “During the most recent grading period, how were your grades?” to examine validity of the PFS-SaNA. Student-report measures were completed in late winter. Schools chose whether students were to complete measures via an Internet-based software system or on paper.

### PFS-SaNA Procedures

During the fall of each year school, personnel were asked to place copies of the PFS-SaNAs in back-to-school packets. If they could not be included in the packets, they were completed during a school open house event. Following parental completion, school staff sorted the PFS-SaNAs. Parents were then invited to participate in a variety of PFS services, such as brief parent groups (e.g., parent topic nights), individual sessions (e.g., Family Check-Up), or meetings to plan and proactively problem solve student supports for the coming year.

### Data Analysis

#### Factor structure

The structure of the PFS-SaNA was assessed in two steps. First, an EFA with a varimax rotation was conducted in SPSS Statistics 19.0 using the 448 PFS-SaNAs collected in 2011–2012 with complete data (i.e., 81.5% of the total number of collected screeners). Results of the EFA were evaluated using standard criteria (e.g., McDermott, 1993). Specifically, the scree plot (Cat-tell, 1966) and total variance accounted for were examined. In addition, the extent to which the solution was meaningful and gleaned a simple structure was considered. Finally, Cronbach's alpha was computed to determine internal reliability with an a priori cutoff value of ≥.80 used to demonstrate adequate reliability. Next, a CFA was conducted in Mplus Version 6.1 (L. K. Muthén & Muthén, 1998–2010) by using the 422 PFS-SaNAs collected in 2012–2013 with complete data (i.e., 85.3% of the total number of collected screeners). Evaluation of the CFA model included assessment of overall goodness of fit, localized areas of misfit in the solution, and interpret-ability, size, and statistical significance of the model's parameters. A weighted least squares estimator was used to account for the categorical nature of the PFS-SaNA data (B. Muthén, 1984). Model fit was assessed based on cutoff values recommended by Hu and Bentler (1999); Tucker–Lewis index (TLI > 0.95; Tucker & Lewis, 1973), comparative fit index (CFI > 0.95; Bentler, 1990), and root mean square error of approximation (RMSEA < 0.06; Steiger & Lind, 1980) were examined.

#### Technical adequacy

To determine the technical adequacy of the PFS-SaNA, Pearson product–moment correlation coefficients (*r*) were compared for the PFS-SaNA and the aforementioned parent- and teacher-report questionnaires. Evaluation of correlation coefficients was based on Cohen's (1988) recommendations for effect size conventions: .2, small; .5, moderate; and .8, large.

## Results

### Factor Structure

#### EFA

Examination of the scree plot and eigenvalues suggested one to two components. Two components had eigenvalues greater than 1.0. A two-factor solution accounted for 57.5% of the variance, but six of the 14 items had cross-loadings greater than 0.30 across the two factors, which suggested a simple structure was not present. Two orthogonal rotations (i.e., equamax and quarti-max) and two oblique rotations (i.e., direct oblimin and promax) were also examined, but a simple structure from these rotations was not gleaned. Thus, a one-factor solution that accounted for 48.8% of the variance was retained. Table 2 includes factor loadings for each of the 14 items for the one-factor solution. Internal consistency was computed and revealed that the PFS-SaNA has good reliability (Cronbach's α = .92).

#### CFA

Examination of CFA fit indices provides additional evidence for the one-factor solution produced from the EFA. Specifically, the CFI (0.97) and TLI (0.96) indicate good fit. The RMSEA (0.08) suggests a marginal fit. In sum, the CFA fit indices suggest an adequate fit for the one-factor solution produced in the EFA. All parameter estimates were statistically significant and positive, which indicates each variable is related to the latent construct. The *r*^2^ values of the observed variables ranged from .47 to .82.

### Technical Adequacy

Table 3 presents descriptive statistics for the scales examined. Table 4 presents correlations between the unidimensional PFS-SaNA and teacher- and parent-report measures. Correlations between scale scores and item-level correlations were examined to provide evidence of validity. Results suggest the PFS-SaNA completed by parents in the fall was correlated with teacher ratings of student behavior in the following spring. In particular, a medium association was observed between the total score on the PFS-SaNA and the TCSSAS. A small to medium association was observed between items on the fall PFS-SaNA and the spring TCSSAS. In addition, findings indicated the fall PFS-SaNA was highly correlated with parent ratings of student behavior in the spring. A large association was noted between the total score on the PFS-SaNA and the total standardized score of adapted TRISK items. A medium to large association was noted between PFS-SaNA items and the corresponding adapted TRISK items.

Examination of initiations of contact with parents by school personnel revealed that parent report of school contact about their student's negative behavior in the spring demonstrated a medium to large correlation (*r* = .43, *p* < .001) with the PFS-SaNA completed the previous fall. These findings suggest that if data from the PFS-SaNA completed in the fall were used by school personnel, it is possible that some of the school contacts about a student's negative behavior the following spring could have been prevented. To provide additional evidence of validity, the PFS-SaNA was correlated with student self-report of grades and spring state standardized math and reading scores. The fall PFS-SaNA was correlated (*r* = .40, *p* < .001) with student self-report of grades in the winter, which suggests that as student grades improve, so does parent report of student functioning. In addition, the fall PFS-SaNA was correlated with state standardized math (*r* = .34, *p* < .001) and reading scores (*r* = .26, *p* < .001).

## Discussion

The purpose of this study was to psychometrically evaluate the PFS-SaNA for use in middle schools. We first review the results of the psychometric findings (i.e., factor structure and technical adequacy) and then discuss implications for use of the PFS-SaNA in middle schools.

### Psychometric Findings

#### Factor structure

Findings from the EFA and CFA suggest items in the PFS-SaNA convey a unidimensional scale that broadly reflects a parent's perception of his or her student's school readiness. This finding is consistent with our hypothesis that despite the multiple domains of functioning assessed by the PFS-SaNA (e.g., classroom, peer relations), a total score can reflect this parental perception. This interpretation is consistent with that of some commercially available screeners (e.g., Behavioral and Emotional Screening System; Kamphaus & Reynolds, 2007). An advantage of the PFS-SaNA is its brevity and positive, engaging wording relevant to concerns parents might have in key domains during the school entry transition (e.g., classroom performance, homework, teacher relationships). In this sense, these findings support the goal of having a psychometrically valid screener at school entry and a tool for increasing the likelihood of engaging parents within a positive behavior support system. The PFS-SaNA is an efficient and pragmatic universal parent assessment for Gate 1 of a multiple-gating protocol that lays the groundwork for immediate proactive engagement or sets the stage for informed second- and third-gate assessments, if needed.

#### Technical adequacy

Results from the factor analysis are encouraging in that they provide necessary evidence for basic psychometric properties of the PFS-SaNA. However, further investigation was warranted to provide sufficient evidence that it was measuring an important construct. To accomplish that, the technical adequacy of the PFS-SaNA was evaluated by examining correlations between it and other scales that assess emotional and behavioral needs of middle school students, as well as assess parent–school contacts.

Findings indicated the fall parent ratings on the PFS-SaNA were correlated with spring parent and teacher ratings of similar student concerns. The PFS-SaNA was more highly correlated with parent ratings of student behavior in the spring than were teacher ratings of student behavior in the spring. A review of item-level correlations indicated items that yield the largest effects are those that ask about students following classroom rules and staying on task in both the PFS-SaNA and the teacher-report measure. Item-level correlations across the PFS-SaNA and parent ratings of students' emotional and behavioral features in the spring indicated the highest correlations were for staying on task in class, completing homework and assignments on time, and getting depressed, anxious, or irritable. These findings suggest that, consistent with our hypothesis, the PFS-SaNA is highly correlated at the scale and the item level with parent and teacher ratings of students' emotional and behavioral symptoms. The observed correlations between the PFS-SaNA and teacher ratings were in the same range as correlations between other parent- and teacher-report measures (e.g., Reynolds & Kamphaus, 2004).

Particularly noteworthy is that the PFS-SaNA completed in the fall was positively correlated with parent report of school-initiated contacts about students' problem behavior the following spring. This finding suggests that the PFS-SaNA may effectively catch students who are struggling early in the school year and may prevent school contacts later in the year after the behavior may have increased in frequency and/or intensity. Furthermore, the PFS-SaNA was positively correlated with state standardized reading and math scores, which provides additional evidence for validity.

### Public Middle School Applications

There are three pragmatic advantages to using a universal assessment strategy during which parents are asked to pro-actively provide information that is not obviously oriented toward mental health or behavioral health. First, proactive screening with parents often provides school staff with permission to establish positive behavior support routines that coordinate resources at home and school (e.g., monitoring) in an effort to prevent difficulties in the coming academic year, without the use of stigmatizing labels. Second, the PFS-SaNA provides school staff with specific behavioral information about incoming students, which can be used when discussing or addressing student concerns. Third, at this stage, the PFS-SaNA functions as a universal assessment. However, the PFS-SaNA may also have utility as a screener, particularly as Gate 1 in a multiple-gating screening process. The PFS-SaNA reverses earlier multiple-gating strategies that began with teachers and proceeded to parents, which in some cases elicited parent resistance to engage with school staff (Dishion & Kavanagh, 2003). For example, Kavanagh, Burraston, Dishion, and Schneiger (2000) found a statistically reliable bias for teachers to inadvertently rate non-White males at higher risk for problem behavior. It is highly likely that proactively engaging these particular parents in a collaborative process to reduce risk would be experienced as offensive.

Despite proactive strategies to encourage a high response rate of parents to complete the PFS-SaNA, some parents will not complete and return it to school. Some may be parents of students who are at risk and may need selected or indicated support. The risk of missing screener data is comparable whether parents or school personnel are used at Gate 1. Stoep et al. (2005) were able to screen only 83% of middle school students by using school personnel at Gate 1. This percentage is in the same range as the response percentage of schools in our study that placed the PFS-SaNA in registration packets (see Table 1).

In addition to proactive efforts to achieve a high response rate from parents and systematic follow-up to obtain screeners from parents who did not initially complete them, other tactics may be used by school personnel to identify student needs. For example, the names of students whose parents did not fill out a PFS-SaNA can be organized so that when data about attendance, work completion, grades, and behavior are first reviewed by school teams in early fall, these students are carefully evaluated with school data to ensure they receive teacher risk screening at Gate 1 or early Gate 2. Furthermore, school staff can continue to reach out and encourage families who have not filled out the PFS-SaNA. Completion rates could be increased by adding incentives for students when their parents return a completed form.

To illustrate how the PFS-SaNA may be used as a screener, screening steps are provided in Figure 1. Before the school year begins, the PFS-SaNA is collected from parents as part of a registration procedure. During the first month of school, before sufficient school data and teacher– student experience are available for the next screening gate, a concerted effort could be made to obtain PFS-SaNAs from the parents of students with missing PFS-SaNAs. Collected screeners could then be sorted by level of parent concern, need for support, and desire to be contacted. By mid-fall, brief teacher screens are completed and school-wide data team review of school data (i.e., behavior, attendance, grades/proficiencies) initiated. Teacher screening data and school data could be used to augment data from PFS-SaNAs. Alternatively, in the event a PFS-SaNA was not completed by parents, teacher screening data and school data could be used instead. Information gleaned from these assessments would inform a tailored and coordinated school-based strategy to respond to student and family needs.

### Limitations and Future Research Directions

Our study findings must be considered in terms of some limitations. First, the sample was derived from those parents who completed the screener and whose data could be linked with other parent- and teacher-report information. Future research would benefit from collecting the PFS-SaNA with a broad spectrum of parents who define the population of a community or district. Second, test–retest data are not currently available for the PFS-SaNA. Future research could examine the retest stability of the measure both within a school year and from one year to the next. However, previous longitudinal research with parent ratings of youth behavior suggests substantial stability over time in perceptions of adjustment (Verhulst, Koot, & Van der Ende, 1994). Third, we were unable to systematically assess the hypothesis that use of the PFS-SaNA would increase the likelihood of parents with high-risk students to engage in evidence-based family interventions. Addressing this question is a promising next step in the overall effort to improve the effectiveness of schools by adding proactive parent engagement within a systemic framework, such as PFS. Fourth, it was not optimal to rely on students' self-report of grades and financial need; therefore, future research should use more objective assessment methods. Finally, it would be particularly important to extend the use of the PFS-SaNA to include assessment of students' emotional health in general, including depression, debilitating anxiety, and/or suicidal ideation.

### Summary

Using a short parent-reported assessment that is psychometrically valid and less stigmatizing at the beginning of the school year may substantially increase the degree of freedom for schools to proactively engage with families before this engagement is contaminated with school-based problems and concerns. This strategy would increase the probability of developing a good working collaboration between home and school that emphasizes the best interests of the student. Moreover, when schools systematically screen and sort according to students' needs, they can more efficiently address those specific needs and promote academic and social–emotional adjustment. By dovetailing the information gained from the PFS-SaNA with an ongoing PBIS structure and tiered support methods, school personnel can offer universal, selected, and indicated interventions specific to needs identified by students' parents (Dishion, 2011). Finally, the PFS-SaNA as a first gate has a low response cost for schools in that teachers do not have to use their time to conduct the screen, and the measure is inexpensive.

## Figures and Tables

**Table 1:** PFS-SaNA Completion and Eligible Students Across Participating Schools.

Academic year	School	School enrollment	PFS-SaNA completed	% completed	Screeners matched to students	Students eligible	Proportion
2011–2012	School 1	359	99	27.58	67	238	.282
	School 2^a^	571	465	81.44	282	377	.748
	School 3^a^	388	337	86.86	201	253	.795
	Total	1,318	901	68.36	550	868	.634
2012–2013	School 1^a^	370	230	62.16	140	236	.593
	School 4	470	266	56.60	78	319	.245
	School 5	457	182	39.82	59	315	.187
	School 6	583	253	43.40	139	348	.399
	School 7	290	129	44.48	34	191	.178
	School 8	756	189	25.00	45	489	.092
	Total	2,926	1,249	42.69	495	1,898	.261

*Note.* In the fall of each academic year, PFS-SaNA data were collected for students at participating schools. All non-PFS-SaNA data used in this study (e.g., teacher-report data) were collected using a rolling cohort data collection scheme. Thus, not all PFS-SaNA data could be matched to other data. PFS-SaNA = *Positive Family Support–Strengths and Needs Assessment*.

a Schools that put the PFS-SaNA in every student's registration packets.

**Table 2:** Factor Structure of the PFS-SaNA.

Items	Factor loadings
Needs structure and supervision to stay on task and	0.81
behave well	
Completes homework and assignments on time	0.80
Focuses and stays on task in class	0.79
Gets easily distracted by other kids	0.78
Follows classroom rules	0.78
Behaves well at school	0.76
Gets grades appropriate for his or her skills	0.74
Cooperates with adults	0.73
Avoids tasks that seem difficult or challenging	0.70
Spends time with students who break school rules	0.62
Has good relationships with other students	0.60
Gets depressed, anxious, or irritable	0.58
Likes attending school	0.55
Shows up on time to school, other activities	0.39

*Note.* Factor structure is based on a random split sample size of 448. PFS-SaNA = *Positive Family Support–Strengths and Needs Assessment*.

**Table 3:** Descriptive Statistics.

Scale	*n*	*M* (*SD*)	Minimum– maximum	Skewness	Kurtosis	Timeframe
PFS-SaNA	448	1.26 (0.33)	1.00–2.93	1.52	2.20	Fall
TCSSAS	319	1.56 (0.76)	1.00–4.00	1.50	1.43	Spring
Adapted TRISK	85	2.89 (1.73)	1.00–8.00	1.17	0.76	Summer
SSRI	85	0.53 (0.52)	0.00–2.33	1.10	0.94	Summer
Negative parent contact	82	0.28 (0.45)	0–1	1.00	1.04	Summer
Positive parent contact	82	0.79 (0.66)	0–3	0.52	0.45	Summer
Socioeconomic status indicator	331	2.60 (0.77)	1.00–4.00	0.27	0.54	Winter
Student-report of grades	339	2.09 (10.20)	1.00–5.00	0.95	0.73	Winter

*Note.* PFS-SaNA = *Positive Family Support–Strengths and Needs Assessment*; TCSSAS = *Teacher Concern for Student School Adjustment Scale*; TRISK = *Teacher Risk Assessment*; SSRI = *Secondary School Readiness Inventory*.

**Table 4:** PFS-SaNA Correlations With Teacher and Parent Measures.

PFS-SaNA	Teacher-report TCSSAS	*r*	*p*
PFS-SaNA total score	TCSSAS total score	.44	<.001
Gets depressed, anxious, or irritable	Depressed, anxious, or irritable	.17	.002
Has good relations with other students	Relations with other students	.28	<.001
Spends time with students who break school rules	Spends time with students who break school rules	.31	<.001
Follows classroom rules	Follows classroom rules	.41	<.001
Focuses and stays on task in class	Stays on task	.35	<.001
Completes homework and assignments on time	Completes homework and assignments on time	.33	<.001

PFS-SaNA	Parent-report measures	*r*	*p*

PFS-SaNA total score	TRISK + SSRI standardized total score	.79	<.001
Gets depressed, anxious, or irritable	Depressed, anxious, or irritable	.53	<.001
Has good relations with other students	Relations with other students	.39	<.001
Spends time with students who break school rules	Spends time with students who break school rules	.50	<.001
Follows classroom rules	Follows classroom rules	.34	.002
Focuses and stays on task in class	Able to focus and stay on task in class	.65	<.001
Completes homework and assignments on time	Completes homework and assignments on time	.61	<.001
Cooperates with adults	Cooperates with adults	.28	.008
Avoids tasks that seem difficult or challenging	Does not avoid tasks that seem difficult or challenging	.58	<.001
Shows up on time to school or other activities	Attends and shows up on time for school or other activities	.21	.057
Behaves well at school	Behaves well at school	.57	<.001
Gets easily distracted by other kids	Does not get distracted by other kids	.54	<.001
Likes attending school	Likes attending school	.55	<.001
Gets grades that are appropriate for his or her skills	Gets grades that are appropriate for his or her skills	.69	<.001

*Note.* PFS-SaNA = *Positive Family Support–Strengths and Needs Assessment*; TCSSAS = *Teacher Concern for Student School Adjustment Scale*; TRISK = *Teacher Risk Assessment*; SSRI = *Secondary School Readiness Inventory*.
